# Optimization of Tissue Culturing and Genetic Transformation Protocol for *Casuarina equisetifolia*

**DOI:** 10.3389/fpls.2021.784566

**Published:** 2022-01-21

**Authors:** Huimin Ren, Yan Xu, Xiaohong Zhao, Yan Zhang, Jamshaid Hussain, Fuqiang Cui, Guoning Qi, Shenkui Liu

**Affiliations:** ^1^State Key Laboratory of Subtropical Silviculture, School of Forestry and Biotechnology, Zhejiang A & F University, Hangzhou, China; ^2^Department of Biotechnology, COMSATS University Islamabad, Abbottabad, Pakistan

**Keywords:** *Casuarina equisetifolia*, regeneration system, transformation system, *Agrobacterium tumefaciens*, *Agrobacterium rhizogenes*

## Abstract

*Casuarina equisetifolia* is widely used in agroforestry plantations for soil stabilization, ecosystem rehabilitation, reclamation, and coastal protection. Moreover, *C. equisetifolia* has remarkable resistance to typhoons, desert, low soil fertility, drought, and salinity, but not cold. Therefore, it is significant to breed high-quality *Casuarina* varieties to improve the tolerance and adaptability to cold weather by molecular techniques. The establishment of a rapid and efficient callus induction and regeneration system *via* tissue culture is pre-requisite for the genetic transformation of *C. equisetifolia*, which is so far lacking. In this study, we reported an efficient and rapid regeneration system using stem segment explants, in which callus induction was found to be optimal in a basal medium supplemented with 0.1 mg⋅L^–1^ TDZ and 0.1 mg⋅L^–1^ NAA, and proliferation in a basal medium containing 0.1 mg⋅L^–1^ TDZ and 0.5 mg⋅L^–1^ 6-BA. For bud regeneration and rooting, the preferred plant growth regulator (PGR) in basal medium was 0.5 mg⋅L^–1^ 6-BA, and a combination of 0.02 mg⋅L^–1^ IBA and 0.4 mg⋅L^–1^ IAA, respectively. We also optimized genetic a transformation protocol using *Agrobacterium tumefaciens* harboring the binary vector pCAMBIA1301 with β-glucuronidase (GUS) as a reporter gene. Consequently, 5 mg L^–1^ hygromycin, 20 mg L^–1^ acetosyringone (As), and 2 days of co-cultivation duration were optimized to improve the transformation efficiency. With these optimized parameters, transgenic plants were obtained in about 4 months. Besides that, *Agrobacterium rhizogenes*-mediated transformation involving adventitious root induction was also optimized. Our findings will not only increase the transformation efficiency but also shorten the time for developing transgenic *C. equisetifolia* plants. Taken together, this pioneer study on tissue culturing and genetic transformation of *C. equisetifolia* will pave the way for further genetic manipulation and functional genomics of *C. equisetifolia*.

## Introduction

*Casuarina equisetifolia*, a symbiotic nitrogen-fixing tree, plays an important role in economic and environmental improvements in tropical and subtropical littoral zones of Asia, the Pacific, and Africa (Diem and Dommergues, 1990; Zhang et al., 2008; Zhong et al., 2010). It is widely used in agroforestry plantations for several purposes, such as soil stabilization, ecosystem rehabilitation, reclamation, and coastal protection (Pinyopusarerk and Williams, 2000). *C. equisetifolia* is also appreciated as a source of fuel wood, paper, timber, medicine, dye, pulp, tannin, and charcoal (Pan et al., 1996; Zhong et al., 2005, 2010; Diagne et al., 2013b; Potgieter et al., 2014).

*Casuarina equisetifolia* is one of the typical angiosperms with distinctive needle-like branchlets, due to which water loss by evapotranspiration is significantly low in *C. equisetifolia*. Therefore, *C. equisetifolia* is a valuable tree to adapt to survival in drought stressed areas (Le et al., 1996). Moreover, it is reported that *C. equisetifolia* establishes symbiotic relationships with soil microorganisms like *Frankia* for nitrogen fixation in *Casuarina* root nodules (Benson and Silvester, 1993; Diagne et al., 2013a) and mycorrhizas for increasing phosphorus uptake (Santi et al., 2003). These symbiotic relationships enhance its adaptability to harsh environments such as salinity, barrenness, and drought.

Since the 1950s, *C. equisetifolia* has been extensively planted in infertile and sandy arid areas along the South China coastline as a windbreak/shelterbelt. Nowadays, it is widely planted, and is one of the most important coastal forest species because of its remarkable resistance to typhoons, desert, low soil fertility, drought, and salinity (Tani and Sasakawa, 2003; Ye et al., 2019). However, low temperature is one of the main limitations for fast growth, cultivation expansion, and high yield of *Casuarina* species (Li et al., 2016). Therefore, it is significant to breed high-quality *Casuarina* varieties to improve its tolerance and adaptability to cold weather using molecular techniques (He et al., 2011). Although few studies have been conducted to understand how *Casuarina* species respond to low temperature at physiological level (He et al., 2011; Wu et al., 2012), little is known about the functional genes and molecular mechanism of cold response in *Casuarina*. Recently, whole genome sequencing of *C. equis. ssp. Incana* has been accomplished. Genome size is reported to be about 300 Mb (Zhong et al., 2005, 2010; Ye et al., 2019). This progress may greatly contribute in studies on molecular mechanisms underlying the phenotype and tolerance to diverse stresses in *C. equisetifolia*. Moreover, it will also pave the way for breeding new varieties by gene function analysis and genetic transformation.

A rapid and efficient callus induction and regeneration system *via* tissue culture is a pre-requisite and essential part for genetic transformation of *C. equisetifolia*, which has not been established so far. Previously, few studies have reported a regeneration and transformation system for *Casuarina glauca Sieb. ex Spreng.* and *Casuarina cunninghamiana Miq.* (Le et al., 1996; Franche et al., 1997; Santi et al., 2003; Obertello et al., 2005). However, owing to great morphological differences among *Casuarina* species (Ye et al., 2019), development of a reliable regeneration and transformation system is crucial to improve and produce new breeds. For plant genetic engineering, *Agrobacterium*-mediated plant transformation has been the most successful and widespread technique in recent decades (Gelvin, 2003b; Vain, 2007). Besides *A. tumefaciens*-mediated transformation, in recent years, *A. rhizogenes*-mediated transformation has been performed to induce adventitious roots, named “hairy roots” at the site of wounding and infection in woody plants (Ozyigit et al., 2013; Hwang et al., 2017).

In this study, we report an efficient, rapid regeneration and two transformation systems for *C. equisetifolia* involving *A. tumefaciens* and *A. rhizogenes*.

## Methods

### Plant Materials and Growth Conditions

Young shoots were cut from 30- to 90-day-old seedlings of *C. equis. ssp. Incana* grown in a greenhouse at 25°C in a 16-h/8-h day/night photoperiod. The shoots were rinsed in running water for 2–3 h and disinfected using 75% ethanol (v/v) for 30 s, followed by rinsing with sterile water five times. Then, the shoots were further disinfected with 6–8% (v/v) sodium hypochlorite for 8 min and rinsed with sterile water five times. Finally, the sterile young shoots were cut into small pieces of about 1 cm in length and placed in a basal medium (1/2 MS, 30 g L^–1^ sucrose and 3.5 g L^–1^ phytagel) supplemented with different phytohormone combinations. The pH of media was adjusted to 5.7. For each hormone treatment, three replicates were performed, each with 14 explants.

For transformation using *A. rhizogenes*, seeds of *C. equisetifolia* were sown in pots filled with soil and grown in the greenhouse at 25°C in a 16-h/8-h day/night photoperiod. After 30 days, healthy seedlings of about 15 cm height were selected for agro-infiltration.

### Optimization of Regeneration

For callus induction, five combinations of plant growth regulators (PGRs) were used as follows: C1: 0.5 mg⋅L^–1^ 6-benzylaminopurine (6-BA) and 0.05 mg L^–1^ kinetin (KT); C2: 1.5 mg⋅L^–1^ 6-BA and 0.03 mg⋅L^–1^ naphthylacetic acid (NAA); C3: 1.5 mg⋅L^–1^ 6-BA and 0.02 IBA; C4: 0.1 mg⋅L^–1^ NAA and 0.1 mg⋅L^–1^ thidiazuron (TDZ); and C5: 0.5 mg⋅L^–1^ 6-BA and 0.1 mg⋅L^–1^ TDZ. Pieces of sterile young shoots were placed in the medium at 28°C in the dark. After about 15 days, the effect of each hormone treatment on callus induction was observed.

For bud regeneration, good-quality calluses were shifted to the media with the following growth regulator combinations: B1: 0.5 mg⋅L^–1^ 6-BA; B2: 0.5 mg⋅L^–1^ 6-BA and 0.05 mg⋅L^–1^ KT; B3: 0.75 mg⋅L^–1^ 6-BA and 0.03 NAA; B4: 1.5 mg⋅L^–1^ 6-BA and 0.02 mg⋅L^–1^ IBA; and B5: 0.5 mg⋅L^–1^ 6-BA and 0.1 mg⋅L^–1^ TDZ. To establish the preferred conditions, the rate of bud regeneration was recorded after growth for 30 days at 28°C under a 16-h light (60–80 μmol photons m^–2^ s^–1^)/8-h dark photoperiod. The buds (about 1 cm in length) were shifted to a rooting medium for root induction. The rate of root induction was calculated after 20-day cultivation in three different rooting media as follows: R1: 0.02 mg⋅L^–1^ IBA and 0.04 mg⋅L^–1^ IAA; R2: 0.4 mg⋅L^–1^ IAA; and R3: 1.5 mg⋅L^–1^ IAA.

### Optimization of Hygromycin Concentration for Transformant Selection

Sterile young stems were placed in an optimized callus induction medium with 250 mg⋅L^–1^ carbenicillin (Car) and six different concentrations of hygromycin (Hyg: 0, 0.5, 1, 3, 5, and 10 mg⋅L^–1^). Each treatment consisted of 20 calluses, and 3 replicates were performed. The status of the calluses was observed, and the rate of callus induction was determined after 15 days of incubation in the dark.

### Vector and Preparation of *Agrobacterium tumefaciens* for Transformation

*Agrobacterium tumefaciens* strain GV3101 was used for genetic transformation of *C. equisetifolia*. The binary expression vector pCAMBIA1301, with a β-glucuronidase (GUS) reporter gene under cauliflower mosaic virus (CaMV) 35S promoter, was mobilized into strain GV3101 using the freeze-thaw method (Holsters et al., 1978). A single colony of *Agrobacteria* carrying the pCAMBIA1301 binary vector was inoculated into an LB liquid containing 25 mg L^–1^ rifampicin and 50 mg L^–1^ kanamycin, and was grown overnight at 28°C with continuous shaking at 220 rpm. A 1-ml culture was transferred to 20-ml fresh liquid LB with appropriate antibiotics and 20 mg L^–1^ acetosyringone (As). The culture was incubated at 28°C until an OD_600_ of 0.6–0.8 was attained, and then centrifugated immediately at 5,000 rpm for 10 min. The pellet was resuspended in an approximately 50-mL ½ MS liquid containing 30 g L^–1^ sucrose and 20 mg L^–1^ As. The final concentration of the *A. tumefaciens* suspension was adjusted to an OD_600_ of 0.6 for transformation.

### Evaluation of Factors Affecting Transformation Efficiency

For transformation, three co-cultivation durations (1, 2, 3 days) and four As concentrations (0, 5, 10, and 20 mg L^–1^) in an *Agrobacterium* infective suspension were evaluated. Stem segments of approximately 1 cm in length each were excised from the sterile young shoots and grown in an optimized callus induction medium. After 15 days, induced calluses were submerged into the *Agrobacteria* infective suspension and shaken at 28°C and 150 rpm for 2 h. Excess bacterial suspensions were removed from the calluses by drying on sterile filter papers. Subsequently, the calluses were placed in a basal medium with 0.1 mg L^–1^ NAA, 0.1 mg L^–1^ TDZ, and different concentration of As (0, 5, 10, and 20 mg L^–1^) for co-cultivation at 28°C. After co-cultivation, the calluses were washed 6–8 times with 250 mg L^–1^ carbenicillin water (each wash was 3 min) and submerged in 250 mg L^–1^ carbenicillin water for 8 min to decontaminate *Agrobacteria*. The induced calluses were then transferred into an optimized selection medium containing PGRs, hygromycin with different concentrations (0, 5, and 10 mg L^–1^), and carbenicillin (250 mg L^–1^). Each treatment consisted of 15 calluses in 3 replicates. All hygromycin-resistant shoots were further assayed by GUS histochemical staining.

### β-Glucuronidase Staining Assay

Various tissues of transgenic young trees and control in different stages of transformation were stained for GUS staining analysis using the method described by Jefferson (Jefferson et al., 1987). After GUS staining, 70% (v/v) ethanol was used to remove the chlorophyll.

### Efficient Hairy Root Transgenic System

A single colony of *Agrobacterium rhizogenes* strain K599 harboring vector pCAMBIA1300-35S:GFP was cultured in 4 ml of LB liquid medium with 50 mg L^–1^ streptomycin and 50 mg L^–1^ kanamycin, and with continuous shaking (180 rpm) at 28°C for about 8–12 h. The inoculum was then transferred to a 20-ml LB broth with the same antibiotics as 1:20. After the OD_600_ reached around 0.8, the cultures were centrifuged at 5,000 × *g* for 10 min at room temperature and then resuspended in 25 ml MES buffer containing 10 mM MES-KOH (pH 5.2), 10 mM MgCl_2_, and 100 μM As.

For transformation, seedlings of 5–8 cm height were gently picked from the pots and the residual soil on the roots was carefully removed. The stem between the cotyledons and roots was gently scratched using a clean blade, and then the entire plant was soaked into the infection solution for 2–4 h. After infection, the seedlings were replanted in the pots for growth. Scratched parts were exposed necessarily above the soil, so callus induction could easily be observed. Callus growth was observed about 15 days after infection in the wounded part of the stem. After about a month, when the hairy roots had developed well, the transgenic rate for the hairy roots was detected using LUYOR-3415RG (Hand-held Lamp). Transgenic efficiency was calculated as:

Transgenic root induction efficiency = (number of plants with induced hairy roots/total number of infected plants) × 100%;

Transformation efficiency = (positive transgenic hairy root lines/total number of induced hairy roots) × 100%.

## Results

### Improvement in Callus Induction and Regeneration of *Casuarina equisetifolia*

**FIGURE 1:**
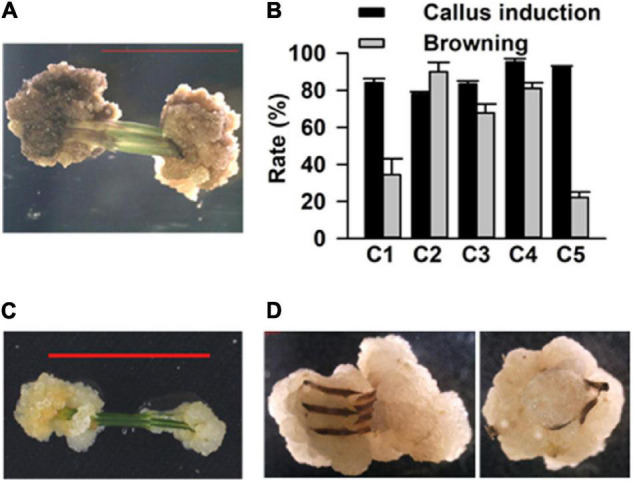
Callus induction of *Casuarina equisetifolia* using the stem as explant. **(A)** Induced callus became brown. **(B)** Callus induction rate and browning callus rate in basal medium with different plant growth regulator (PGR) combinations. The results are presented as the mean and standard error from three independent experiments. **(C)** Inducement and **(D)** proliferation of calluses.

To establish a simple, rapid, and efficient regeneration system for *C. equisetifolia*, we tested 38 different combinations of plant growth regulators (PGR, cytokinin 6-BA, KT, TDZ, auxin IAA, IBA, an NAA) in a basal medium (1/2 MS medium, 30 g L^–1^ sucrose and 3.5 g L^–1^ phytagel) (Supplementary Table 1). Stem segments of approximately 1 cm in length each were excised from the sterile young shoots and grown in the abovementioned medium to induce callus (Supplementary Figure 1). After 15 days of growth in the dark in these media, five combinations exhibiting higher callus induction rate (over 80%) were selected for further experiments. The selected media were renamed as C1 (NO. 30: 0.5 mg⋅L^–1^ 6-BA + 0.05 mg⋅L^–1^ KT), C2 (NO. 6: 1.5 mg⋅L^–1^ 6-BA + 0.03 mg⋅L^–1^ NAA), C3 (NO. 26: 1.5 mg⋅L^–1^ 6-BA + 0.02 mg⋅L^–1^ IBA), C4 (NO. 37: 0.1 mg⋅L^–1^ TDZ + 0.1 mg⋅L^–1^ NAA), and C5 (NO. 32: 0.1 mg⋅L^–1^ TDZ + 0.5 mg⋅L^–1^ 6-BA). It was later observed that the calluses turned brown in the C2, C3, and C4 media (Figure 1A). The statistical results showed that the rate of callus induction was highest (around 98%) in the C4 medium, but that the frequency of callus browning was also higher in the C4 medium compared to that in the others (Figure 1B). On the contrary, the calluses in C5 had lower browning rate (Figure 1B). Thus, the C4 medium was used to induce calluses, while C5 was used for callus proliferation (Figures 1C,D).

**FIGURE 2:**
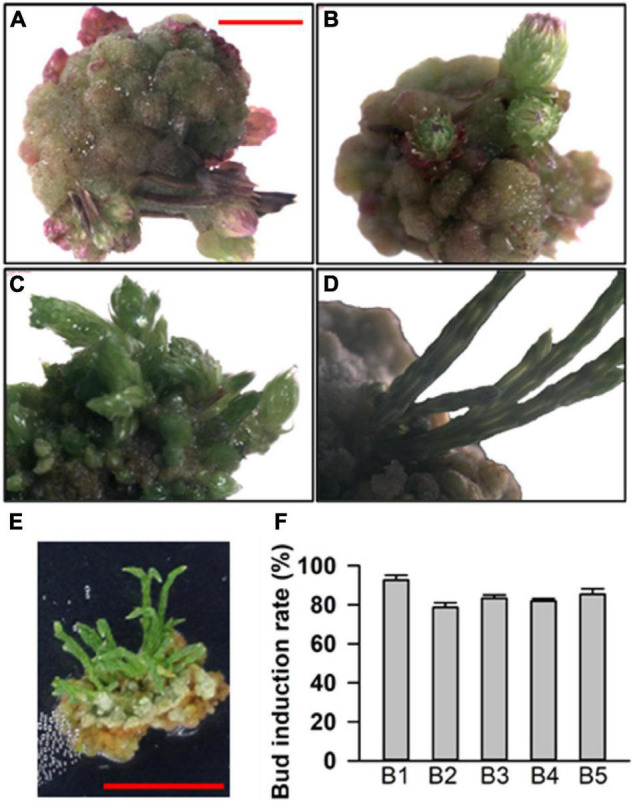
Adventitious bud induction of callus. **(A–D)** Different stages of induced adventitious bud growth. **(E)** Adventitious buds on a callus. **(F)** Bud induction rate in basal medium with different plant growth regulator (PGR) combinations. The results are presented as the mean and standard error from three independent experiments.

For budding induction, we found that budding can be successfully induced in medium numbers 7, 30, 5, 26, and 32, which were renamed as B1 (0.5 mg⋅L^–1^ 6-BA), B2 (0.5 mg⋅L^–1^ 6-BA + 0.05 mg⋅L^–1^ KT), B3 (0.75 mg⋅L^–1^ 6-BA + 0.03 mg⋅L^–1^ NAA), B4 (1.5 mg⋅L^–1^ 6-BA + 0.02 mg⋅L^–1^ IBA), and B5 (0.5 mg⋅L^–1^ 6-BA + 0.1 mg⋅L^–1^ TDZ). Besides B1, which only contained 6-BA, the other media had 6-BA with KT, NAA, IBA, and TDZ. It took about 1–2 months for the calluses to develop adventitious buds (Figures 2A–E). With respect to the rate of budding induction, the B1 medium was found to be the best one (Figure 2F), indicating that only 6-BA was sufficient for budding induction while other hormones did not seem to play any obvious role. To sum it up, the basal medium supplemented with 0.5 mg⋅L^–1^ 6-BA was found to be suitable for budding induction.

**FIGURE 3:**
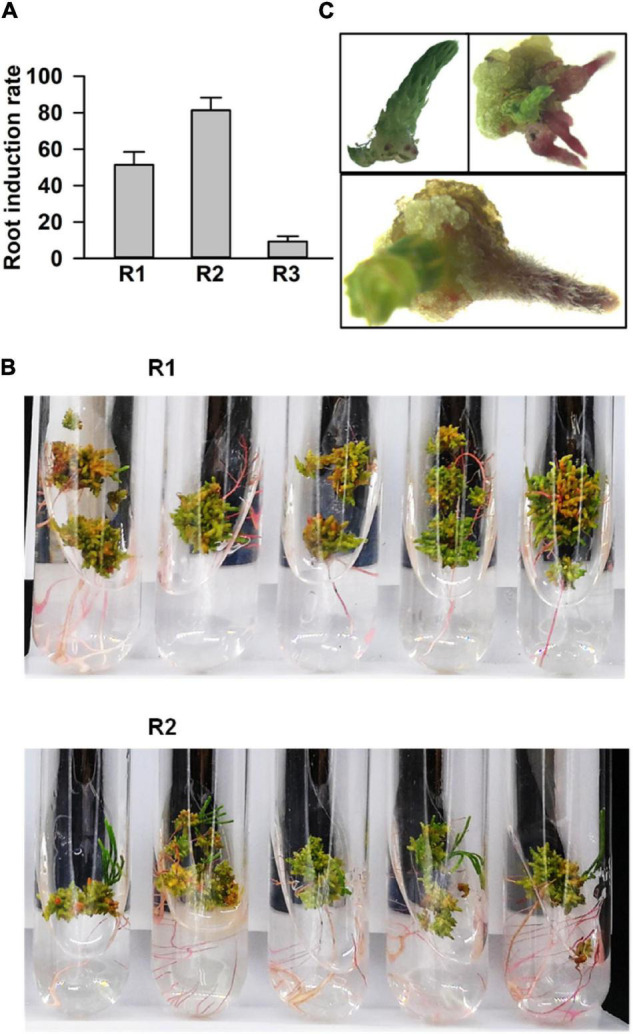
Root regeneration of adventitious buds. **(A)** Root induction rate in basal medium with different plant growth regulator (PGR) combinations. The results are presented as the mean and standard error from three independent experiments. **(B)** Comparing roots in the R1 and R2 media. **(C)** Different stages of induced root.

**FIGURE 4:**
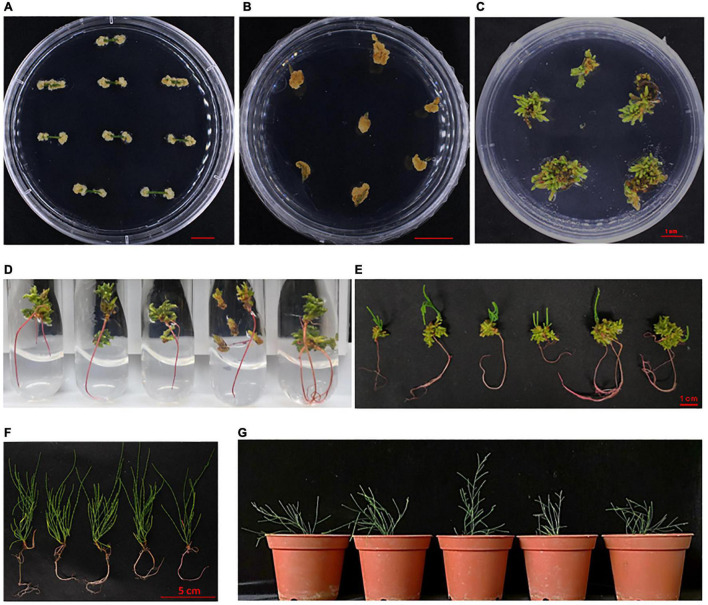
Efficient regenerating system for *Casuarina equisetifolia* using the stem as explant. **(A)** Callus induction in the C4 medium. **(B)** Callus proliferation in the C5 medium. **(C)** Bud induction in the B14 medium. **(D)** Root induction in the R1 medium. **(E)** Root regeneration of adventitious bud. **(F)** Seedlings using suitable regenerating system. **(G)** Transplantation of regenerated seedling.

Next, we processed the buds for root regeneration. When the length of regenerated buds was more than 1 cm, these were divided and transferred to the rooting media. Among 38 tested combinations of growth hormones, 3 media (Nos. 20, 21, and 22) significantly promoted multiple root organogenesis. The statistical analysis of root induction rate showed that the rate in R2 (NO. 21: 0.02 mg⋅L^–1^ IBA + 0.4 mg⋅L^–1^ IAA) was 80%, while in R1 (NO. 20: 0.02 mg⋅L^–1^ IBA + 0.04 mg⋅L^–1^ IAA), and R3 (NO. 22: 0.02 mg⋅L^–1^ IBA + 1.5 mg⋅L^–1^ IAA) it was around 55 and 10%, respectively (Figure 3A). The number of induced roots in R2 was much higher than that in the R1 medium (Figure 3B). Roots emerged within a week after transfer in the rooting medium (Figure 3C). Finally, the whole seedlings, developed through tissue culture, were transferred into a basal medium supplemented with 0.5 g⋅L^–1^ activated carbon to accelerate and improve the quality of rooting (Zhao, 1999; Xu et al., 2006). Taken together, the regeneration of *C. equisetifolia* from explant to fully developed plantlet was accomplished in about 4 months, a greatly shortened duration for *C. equisetifolia* growth (Figure 4).

**FIGURE 5:**
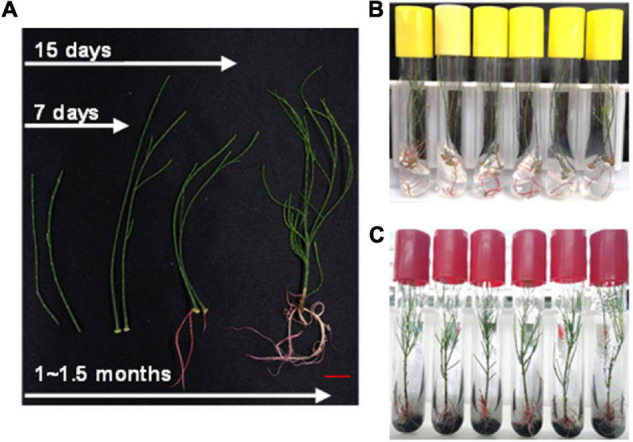
Regenerating system for *Casuarina equisetifolia* by cutting. **(A)** Whole process of regenerating system by cutting. **(B)** Rooting of branchlet in the preferred root medium. **(C)** Branchlets with young roots were transferred to the basal medium supplemented with activated carbon.

In addition to the above method, stem cuttings from aseptic seedling were also used for rapid regeneration of plants. The 8–10 cm long branchlets were cut and transferred to the rooting medium (also supplemented with activated carbon). It took about 7–10 days to generate calluses and 15 days to develop roots (Figure 5A). Then, these were transferred to the basal medium supplemented with activated carbon (Figures 5B,C). The whole process of regeneration from stem cuttings took only about 45 days (Figure 5A). The rate of rooting from cutting was up to 90% (data not shown). This method further shortened the growth cycle of *C. equisetifolia* to a large extent and was, therefore, an important achievement keeping in view plant transformation and subsequent regeneration.

### Determination of Hygromycin and Carbenicillin Concentration for the Selection of Transformants

**FIGURE 6:**
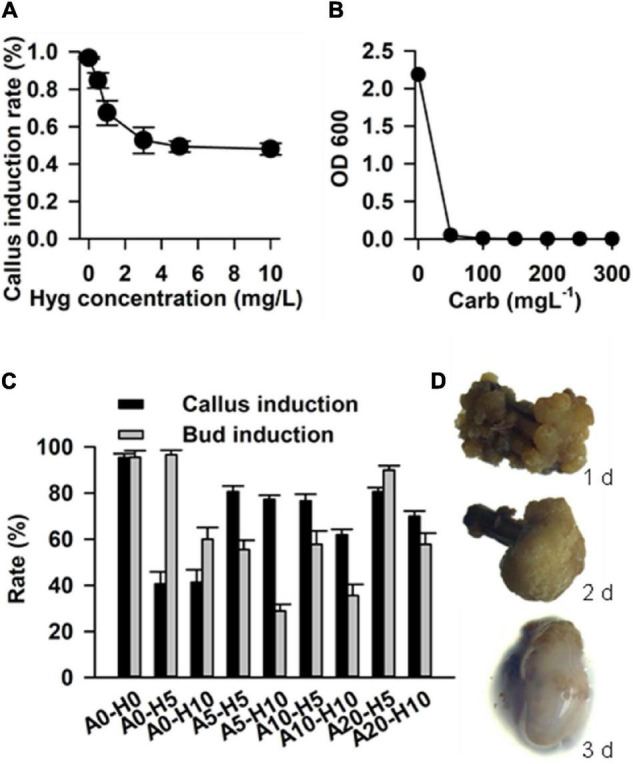
Factors that affect transformation efficiency in *Casuarina equisetifolia*. **(A)** Hygromycin concentration for the selection of transformants. **(B)** Carbenicillin concentration for the inhibition of *Agrobacterium*. **(C)** Rate of callus and bud induction in the medium with different concentrations of acetosyringone (As) and hygromycin. The results are presented as the mean and standard error from three independent experiments. **(D)** Co-cultivation duration for transformation frequency. A0, A5, A10, and A20: concentration of As are 0, 5, 10, and 20 mg/L; H0, H5, and H10: concentration of hygromycin were 0, 5, and 10 mg/L.

In order to determine the effective concentration for antibiotic selection, stem segment explants were grown in the callus induction medium supplemented with a series of hygromycin concentrations (0.5, 1, 3, 5, and 10 mg L^–1^). The results showed that the rate of callus induction declined with increasing in concentration of hygromycin, and reached the steady state at hygromycin a concentration of 5 mg L^–1^ (Figure 6A). Hence, 5 mg L^–1^ was determined to be optimum concentration of hygromycin for the selection of transgenic calluses.

In addition, we tested different concentrations of carbenicillin (Car), which was required to remove residual *Agrobacterium* GV3101 from the calluses. Various concentrations of carbenicillin (50, 100, 150, 200, 250, and 300 mg L^–1^) were supplemented into the *Agrobacterium* liquid medium for callus growth. We found that 100 mg L^–1^ carbenicillin was effective in inhibiting *Agrobacterium* growth (Figure 6B). In the case of stem explants, 250 mg L^–1^ carbenicillin was used to inhibit *Agrobacterium* without any effect on callus proliferation and bud regeneration.

### Effect of Acetosyringone on Transformation

Acetosyringone is an inducer that activates Virgene on Ti plasmid and regulates T-DNA transfer during *Agrobacterium*-mediated plant transformation (Turk et al., 1994). It is useful to add As into a cocultivation medium for improving transformation efficiency (Rashid et al., 2011). In our study, we tested the callus induction rate and budding induction rate in a selected medium supplemented with different concentrations of As and hygromycin, including As 0 + Hyg 0 (without As and hygromycin, as control), As 0 + Hgy 5 mg L^–1^, As 0 + Hgy 10 mg L^–1^, As 5 mg L^–1^ + Hgy 5 mg L^–1^, As 5 mg L^–1^ + Hgy 10 mg L^–1^, As 10 mg L^–1^ + Hgy 5 mg L^–1^, As 10 mg L^–1^ + Hgy 10 mg L^–1^, As 20 mg L^–1^ + Hgy 5 mg L^–1^, and As 20 mg L^–1^ + Hgy 10 mg L^–1^. Compared with the control and other combinations, the combination with As 20 mg L^–1^ and Hgy 5 mg L^–1^ was found to be the best for transgenic callus and budding induction (Figure 6C).

### Determination of Co-cultivation Duration

In addition to the above-mentioned factors, co-cultivation duration (1–3 days), was also optimized to improve the frequency of transformation. *Agrobacterium* GV3101 harboring the vector pCAMBIA1301 (with GUS as a reporter gene) was used for transformation. We found that up to 80% of the calluses were surrounded by *Agrobacterium* after 3 days of co-cultivation, and that most of the calluses grew well after 1 or 2 days (Figure 6D). Therefore, 2 days of co-cultivation was determined to be suitable for *C. equisetifolia* transformation.

### Confirmation of Transgenic Plants by β-Glucuronidase Staining and Reverse Transcription-PCR

**FIGURE 7:**
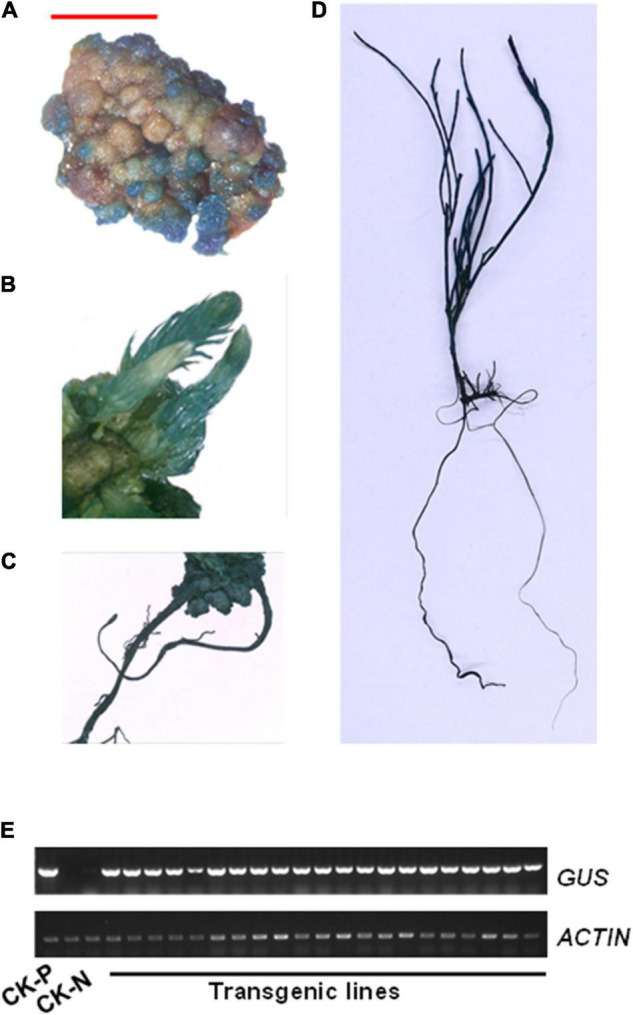
β-Glucuronidase (GUS) expression in transgenic *Casuarina equisetifolia* plants. **(A–D)** GUS staining was observed in the **(A)** callus, **(B)** adventitious bud, **(C)** root, and **(D)** transgenic plantlet. **(E)** Analysis of GUS transcriptional levels in pCAMBIA1301 plasmid (P), non-transformed plants (N) and transgenic lines by RT-PCR analysis.

To confirm the positive transgenic plants, hygromycin resistant tissues in different stages, such as callus, bud, root, and seedling, were stained using a GUS buffer solution. A strong GUS staining signal was detected in different tissues of transgenic plants, but not in those of control plants (Figures 7A–D and Supplementary Figure 2). Furthermore, gene presence was also confirmed by reverse transcription-PCR (RT-PCR) amplification of the GUS reporter gene using GUS-specific primers and genomic DNA as template. A 625-bp band was detected only in transgenic lines but not in untransformed control plants (Figure 7E). These data confirmed the successful integration and expression of a foreign gene into the genome of *C. equisetifolia*.

**FIGURE 8:**
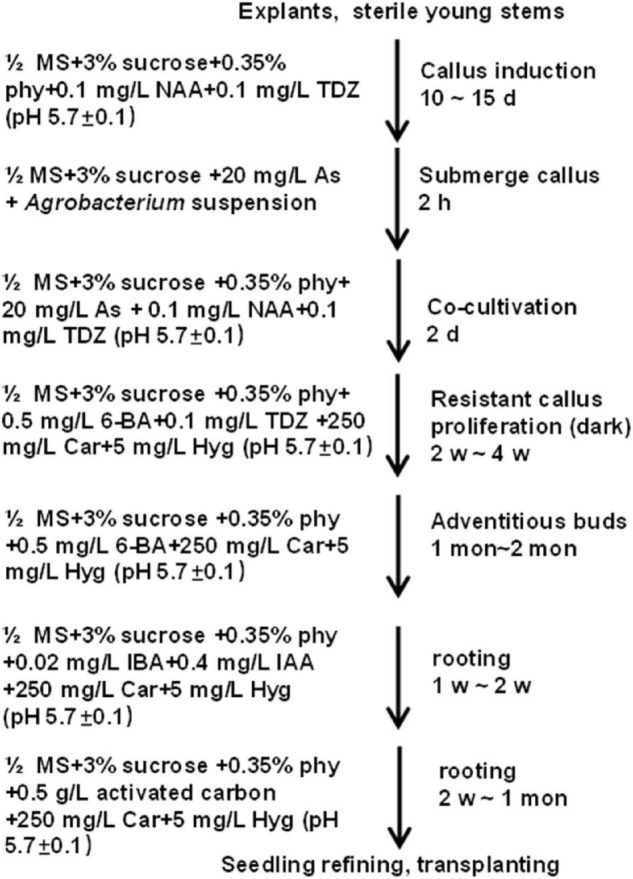
Stepwise protocol for transforming *Casuarina equisetifolia* using stem segments as the explant.

In summary, to get transgenic plants, explants were used to induce calluses in the C4 medium for 10–15 days at 28°C in the dark. Then, good-quality calluses were infected with *Agrobacterium* (GV3101) carrying the CaMV 35S:GUS binary vector at OD_600_ ≈0.8 for 2 h and co-cultivated in a co-cultivation medium for 2 days. Next, the infected calluses were transferred to a medium containing 0.5 mg L^–1^ 6-BA, 0.1 mg L^–1^ TDZ, 250 mg L^–1^ Car, and 5 mg L^–1^ Hyg for resistant callus proliferation. After 2–4 weeks, a selection medium with 0.5 mg L^–1^ 6-BA, 250 mg L^–1^ Car, and 5 mg L−^1^ Hyg was used for adventitious bud growth, which may last for 1–2 month. Afterward, buds with more than 1-cm length were shifted to the rooting medium supplemented with 0.02 mg L^–1^ IBA, 0.4 mg L^–1^ IAA, 250 mg L^–1^ Car, and 5 mg L^–1^ Hyg. Roots appeared in about 1–2 weeks after shifting the buds to the rooting medium. For better growth, the seedlings were planted into the basal medium supplemented with 0.5 g L^–1^ activated carbon, 5 mg L^–1^ Hyg and 250 mg L^–1^ Car to improve root growth (Figure 8 and Supplementary Table 2). When the height of transgenic plantlets reached about 10 cm, they were transplanted in the greenhouse at 28°C with an 8-h light/16-h dark photocycle. Besides, cuttings were also used for rapid regeneration of the transgenic plants.

### Establishment of a Transgenic System for *Casuarina equisetifolia* Through Hairy Roots

**FIGURE 9:**
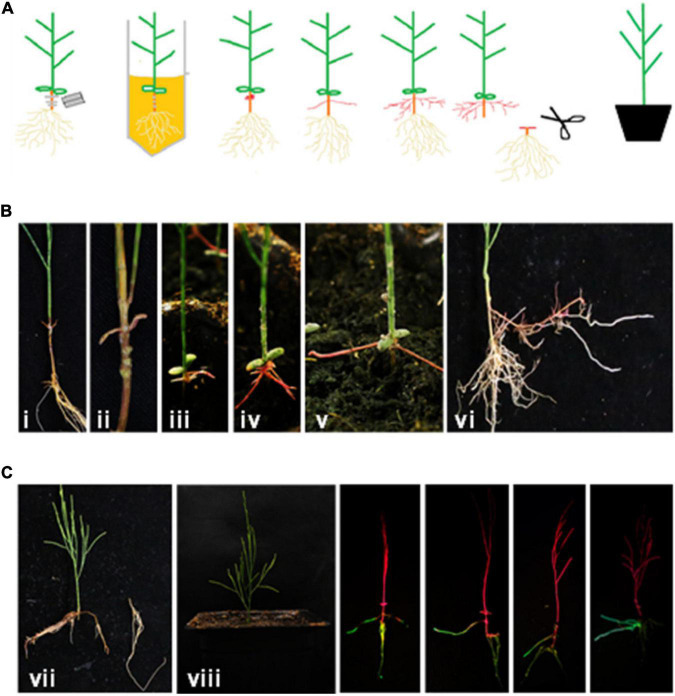
Hairy root transgenic system in *Casuarina equisetifolia*. **(A)** Schematic diagram of transgenic system for *C. equisetifolia* by *Agrobacterium rhizogenes-*mediated transformation. **(B)** Process of callus and hairy root regeneration. (i) The 5–8 cm height seedlings for infection; (ii–vii) different stages of callus and hairy root regeneration. (vi) The picture before cutting off the original root, and (vii) the picture of cutting off the original root after transgenic hairy roots fully developed; (viii) seedling with fully developed transgenic hairy roots. **(C)** GFP signal in transgenic hairy roots.

An *Agrobacterium*-mediated transformation system has been the most successful and widespread method for plant genetic engineering in recent decades (Gelvin, 2003a; Vain, 2007; Matveeva and Lutova, 2014). An efficient root transgenic system has previously been reported for the major woody food crop pigeon pea (Meng et al., 2019). Owing to the superior abiotic stress tolerance of *C. equisetifolia*, we deemed it necessary to establish a fast, simple, and efficient root transgenic method for this plant. To do this, the vector pCAMBIA1-35S:GFP was introduced into *A. rhizogenes* strain K599 or C58C1. As shown in Table 1, *A. rhizogenes* strain K599 exhibited much higher efficiency in transgenic root induction (28.3%) and transformation (95.9%) than strain C58C1 (0%). The schematic diagram of the transgenic system for *C. equisetifolia* by *A. rhizogenes*-mediated transformation is shown in Figure 9A. *C. equisetifolia* seedlings of 5–8 cm height were scratched and infected using an *Agrobacterium* suspension (Figure 9Bi). After about 2 weeks of growth, obvious callus growth was observed around the wound site which then gradually expanded (Figure 9Bii). About 2 weeks later, small hairy roots grew from the calluses (Figures 9Biii–vi). Then, the original roots were cut off after transgenic hairy roots fully developed, and the seedlings were transplanted in a pot (Figures 9Bvii,viii). Furthermore, a strong GFP signal was detected in most of the transgenic roots (Figure 9C). It indicated that the GFP gene was successfully transformed into the hairy root of *C. equisetifolia*. Overall, it took about 2 months to generate the transgenic plants. The transformation protocol optimized in this study would be greatly helpful in studying the function of key stress-responsive genes in *C. equisetifolia.*

**TABLE 1:** Transgenic hairy roots of *Casuarina equisetifolia* using dipping cut stem in bacterial suspension.

	Total number of infected plants	Number of plants with induced hairy root	Transgenic root induction efficiency	Positive transgenic hairy root lines	Total number of induced hairy root	Transformation efficiency
K599	145	41	28.3%	118	123	95.9%
C58C1	153	0	0	0	0	0

## Discussion

Due to its potential for revegetation under arid conditions, *C. equisetifolia* has got considerable attention for research. Moreover, its whole genome has recently been sequenced (Ye et al., 2019). Therefore, it is important to develop a simple, rapid, and efficient genetic transformation system for *C. equisetifolia*, so the function of its key genes *in vivo* could be studied, especially under stress conditions such as drought, cold, and saline-alkali. Although some *Casuarina* species, such as *C. glauca* and *Allocasuarina verticillata* Lam. have been transformed successfully (Le et al., 1996; Franche et al., 1997; Santi et al., 2003), differences in these species limit the application of the protocols for the transformation of *C. equisetifolia*, which leads to impediment in the utilization of its genomic resources. Although it has been reported that GUS or GFP expression was observed in *C. equisetifolia* calluses and young shoots, rooted transgenic plants were not obtained successfully (Zhong et al., 2011). In this study, a novel regeneration and two efficient transformation systems were successfully developed for *C. equisetifolia*. From the explants to the development of transgenic plants using a *A. tumefaciens*-mediated (GV3101) method, it took about 4.5 months in total. On the other hand, plant transformation using the *Agrobacterium rhizogenes*-mediated (K599) hairy root development system took around 45 days to generate transgenic plants, which fairly shortened the time to produce transgenic plants and saved time for studying tree biology at the molecular level.

### Plant Growth Regulators Exhibit Significant Effects on the Efficiency of Regeneration

Plant growth regulators not only perform a significant role in plant developmental processes but also play an important part in plant tissue culture. To establish an efficient regeneration system for *C. equisetifolia*, different kinds of auxin or cytokinin, such as NAA, IAA, IBA, 6-BA, TDZ, and KT, and various combinations of those were used in the basal medium. In this study, we found that cytokinin TDZ was more effective because of its higher callus induction efficiency (Figure 1B). Moreover, TDZ, in combination with 6-BA, could significantly decrease the rate of callus browning compared to its combination with NAA (Figure 1B). The best condition for adventitious bud induction in our study was the basal medium containing only 0.5 mg L^–1^ 6-BA, in which bud induction rate exceeded 95% (Figure 2F). IBA, a main auxin, is widely used to induce adventitious roots in many plant species (Srivastava, 2002; Ludwig-Müller et al., 2005). In our research, the basal medium with 0.02 mg L^–1^ IBA and 0.4 mg L^–1^ IAA was found to be effective in *C. equisetifolia* root induction (Figures 3A,B).

Following genetic transformation with the strain GV3101 harboring vector pCAMBIA1301, several factors were evaluated and optimized in our study. In previous studies, 200–250 mg/L cef has been used (Le et al., 1996; Franche et al., 1997; Santi et al., 2003). Here, 250 mg L^–1^ Car was added into the selection medium rather than Cef. This is because the green adventitious buds turned to brown after growth of about 1 month in the selection medium with Cef (250 mg L^–1^) (data not shown). Co-culture duration is also an important factor altering transformation efficiency by influencing *Agrobacterium*-plant cell interactions, and transformation efficiency for different species benefit from different co-cultivation (Le et al., 1996; Li et al., 2017; Song et al., 2020). In this study, 2 days of co-cultivation was found to be the best for infected callus growth to improve transformation efficiency (Figure 6D).

Acetosyringone, a phenolic compound, induces *vir* gene expression and promotes the efficiency of plant genetic transformation (Stachel et al., 1985, 1986; Nonaka et al., 2008). Our results demonstrated that the addition of As into the *Agrobacterium* suspension and co-cultivation medium obviously improved the rate of callus and bud induction (Figure 6C). The development of an efficient genetic transformation system will facilitate physiological and molecular biology studies on *C. equisetifolia*. Thus, it is important to shorten the time required for the development of *C. equisetifolia* transgenic plants. *A. rhizogenes*-mediated root transformation not only saves time for transgenic plant production, but also provides an efficient way to assess gene function. As each transgenic root originates from a single cell in *A. rhizogenes*-mediated root transformation, a great number of transformants can be obtained (Bercetche et al., 1987; Choi et al., 2004) and analyzed in a relatively short period of time (Kereszt et al., 2007). The *Agrobacterium* strain is one of the factors that influence the process of T-DNA delivery from *Agrobacterium* into plant cells (Crane et al., 2006). Again, transgenic root induction efficiency and transformation efficiency using K599 reached up to 28.3 and 95.9%,which were much higher than those using C58C1 (Table 1). Moreover, it took just about 45 days to obtain the transgenic plants. Thus, this system was highly efficient and convenient for abundant production of transgenic *C. equisetifolia* plants in a short time.

In summary, we established and optimized the protocol for regeneration and two different systems for genetic transformation of *C. equisetifolia*. Because of higher efficiency and simple propagation, these transformation systems can be used to produce *C. equisetifolia* transgenic lines with a variety of genes, which may be conductive to study its unique traits, especially tolerance to drought, salinity, and saline-alkali. Moreover, the establishment of these systems could make it possible to improve its cold stress tolerance by genetic engineering.

## Data Availability Statement

The original contributions presented in the study are included in the article/Supplementary Material, further inquiries can be directed to the corresponding authors.

## Author Contributions

HR: conceptualization, methodology, formal analysis, investigation, writing—original draft, review and editing, and funding acquisition. YX: methodology, formal analysis, investigation, and writing—review and editing. XZ: methodology, formal analysis, and investigation. YZ: methodology and formal analysis. JH: writing—review and editing. FC: investigation. GQ and SL: conceptualization, writing—review and editing, and funding acquisition. All authors contributed to the article and approved the submitted version.

## Conflict of Interest

The authors declare that the research was conducted in the absence of any commercial or financial relationships that could be construed as a potential conflict of interest.

## Publisher’s Note

All claims expressed in this article are solely those of the authors and do not necessarily represent those of their affiliated organizations, or those of the publisher, the editors and the reviewers. Any product that may be evaluated in this article, or claim that may be made by its manufacturer, is not guaranteed or endorsed by the publisher.
