# Supplementary material for: Optimization of Tissue Culturing and Genetic Transformation Protocol for Casuarina equisetifolia
Source: Front Plant Sci. 2022 Jan 21;12:784566. doi: 10.3389/fpls.2021.784566 (PMC8814579; doi:10.3389/fpls.2021.784566)
Supplement: Supplementary file 1 [file Presentation_1.PPTX]

## Slide 1
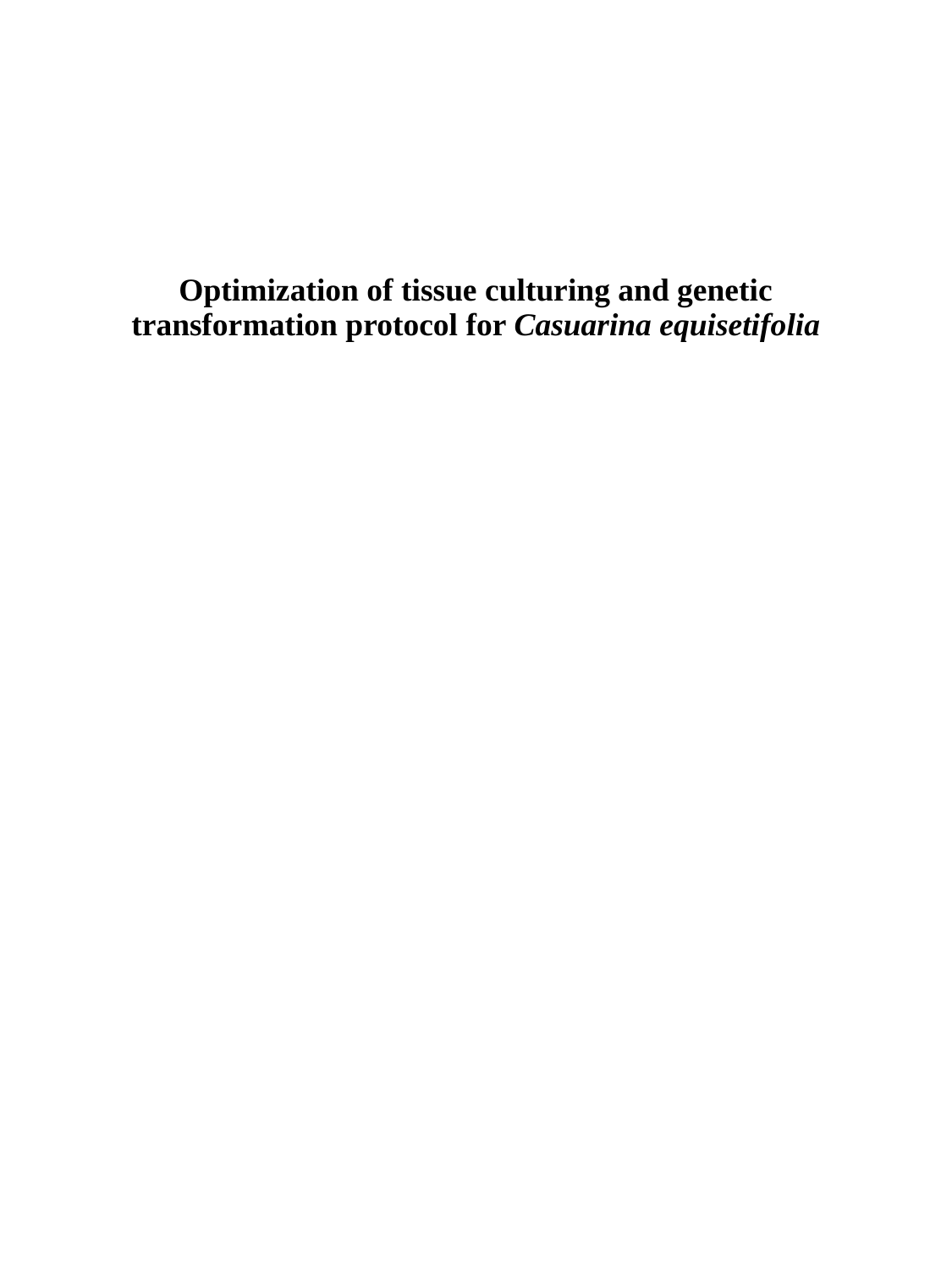

# Optimization of tissue culturing and genetic transformation protocol for Casuarina equisetifolia

## Slide 2
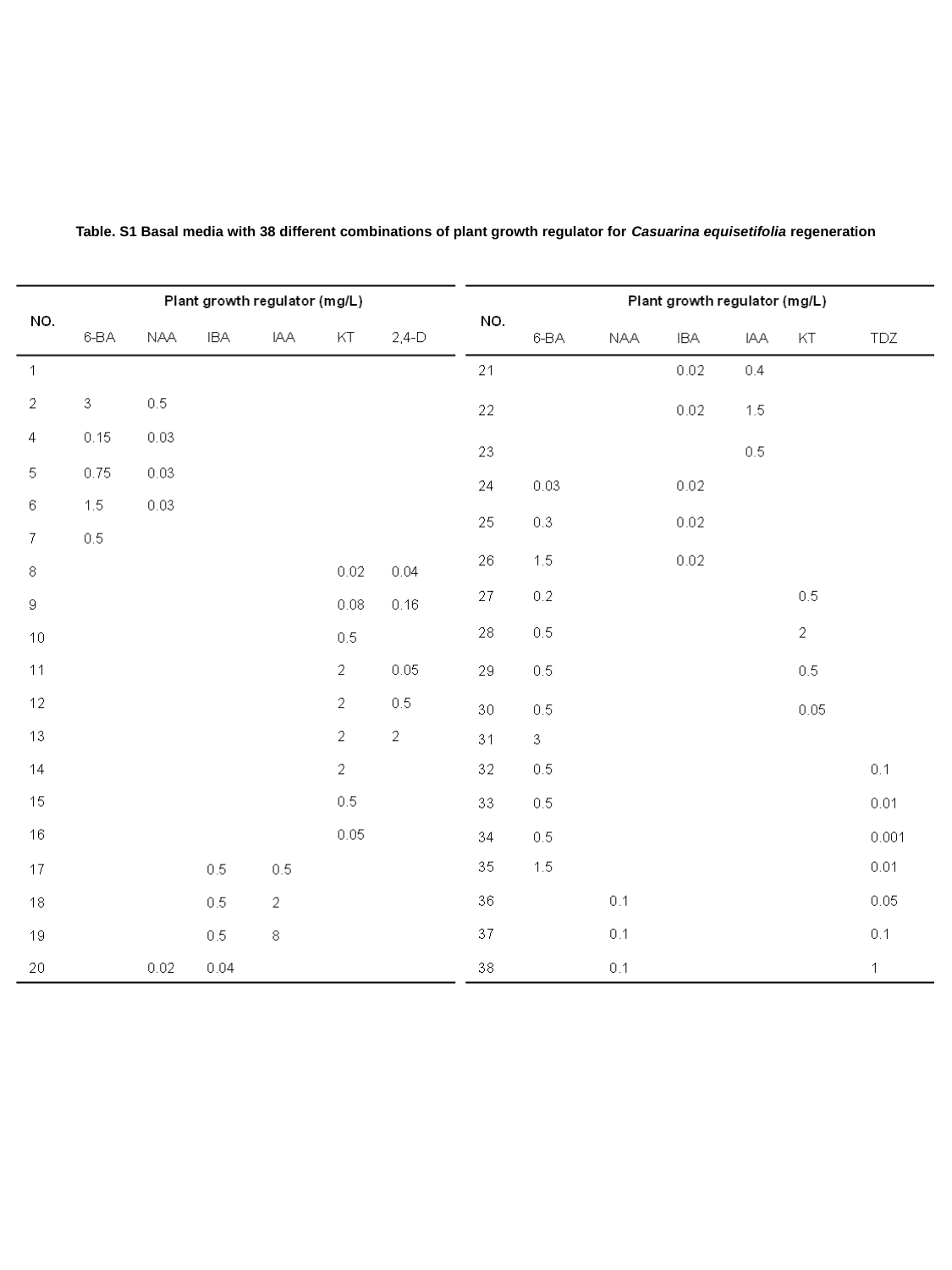

Table. S1 Basal media with 38 different combinations of plant growth regulator for Casuarina equisetifolia regeneration

## Slide 3
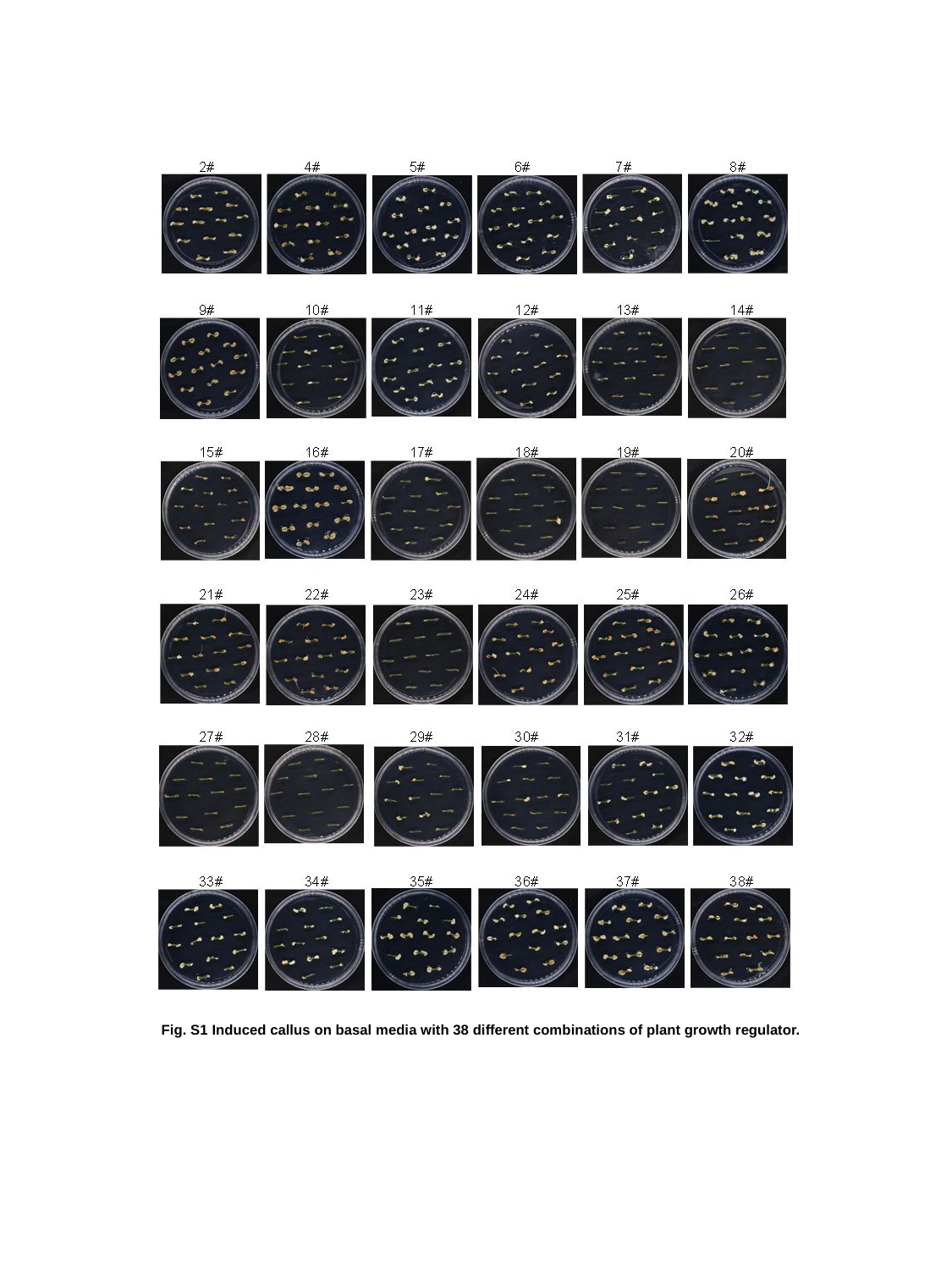

Fig. S1 Induced callus on basal media with 38 different combinations of plant growth regulator.

## Slide 4
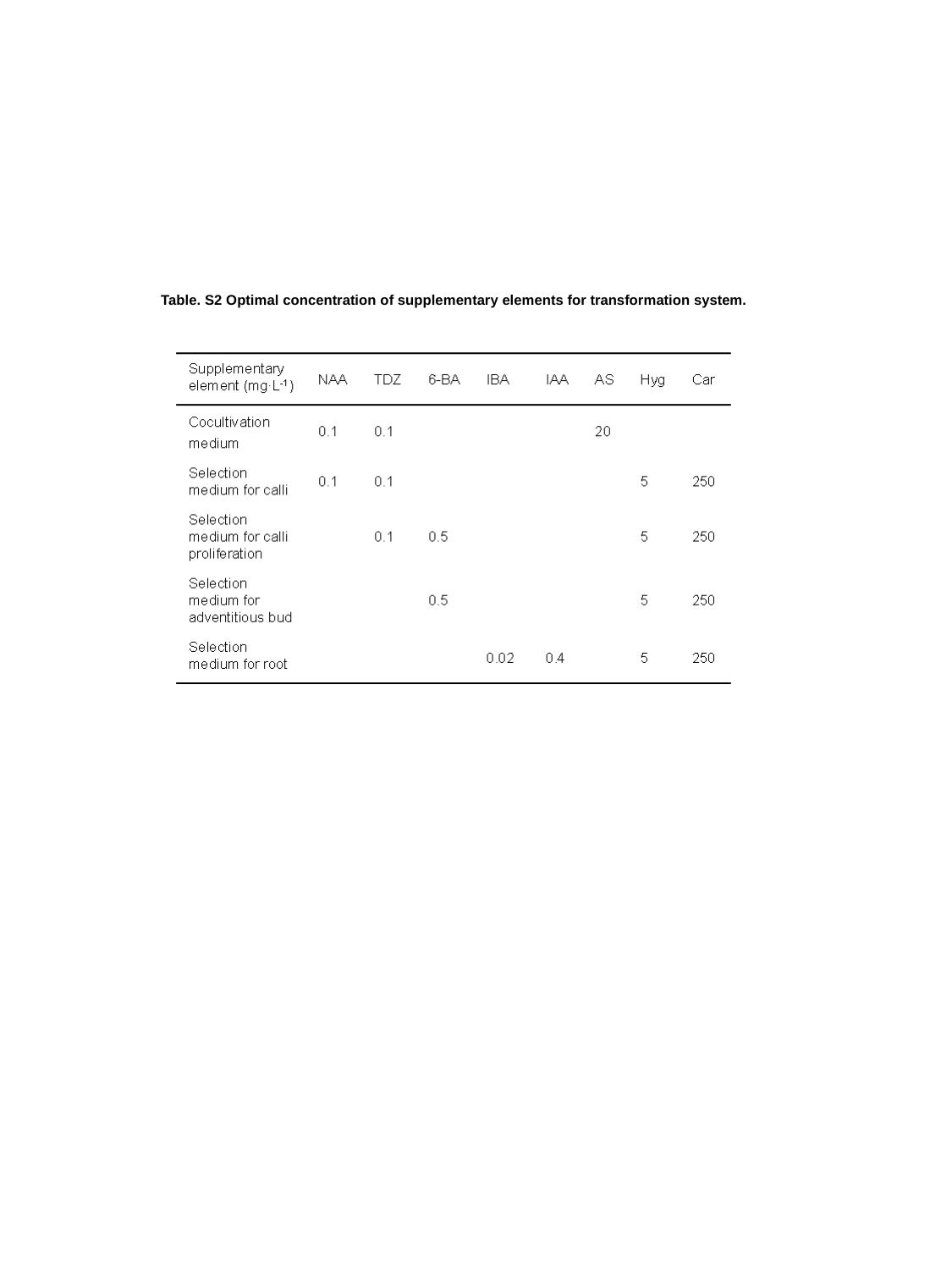

Table. S2 Optimal concentration of supplementary elements for transformation system.

## Slide 5
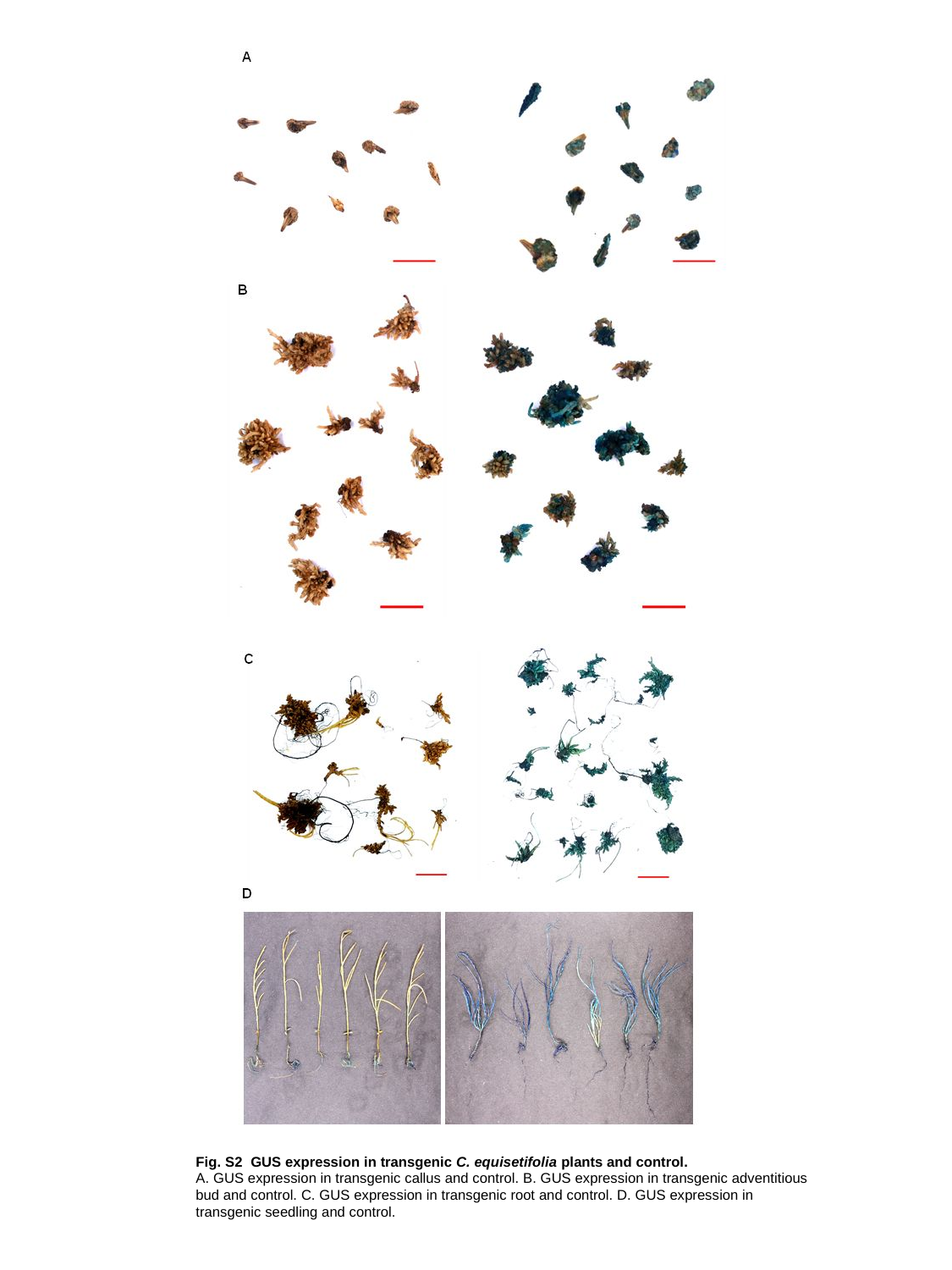

Fig. S2 GUS expression in transgenic C. equisetifolia plants and control.
A. GUS expression in transgenic callus and control. B. GUS expression in transgenic adventitious bud and control. C. GUS expression in transgenic root and control. D. GUS expression in transgenic seedling and control.
